# Numbat-multiome: inferring copy number variations by combining RNA and chromatin accessibility information from single-cell data

**DOI:** 10.1093/bib/bbaf516

**Published:** 2025-10-17

**Authors:** Ruitong Li, Jean-Baptiste Alberge, Tina Keshavarzian, Junko Tsuji, Johan Gustafsson, Mahshid Rahmat, Elizabeth D Lightbody, Stephanie L Deng, Santiago Riviero, Mendy Miller, F Naz Cemre Kalayci, Adrian Wiestner, Clare Sun, Mathieu Lupien, Irene Ghobrial, Erin Parry, Teng Gao, Gad Getz

**Affiliations:** Harvard Medical School, 25 Shattuck Street, Boston, MA 02115, United States; Broad Institute of MIT and Harvard, 415 Main Street, Cambridge, Cambridge, MA 02142, United States; Harvard Medical School, 25 Shattuck Street, Boston, MA 02115, United States; Broad Institute of MIT and Harvard, 415 Main Street, Cambridge, Cambridge, MA 02142, United States; Department of Medical Oncology, Dana-Farber Cancer Institute, 450 Brookline Avenue, Boston, MA 02115, United States; Department of Medical Biophysics, University of Toronto, 101 College Street, Toronto, ON M5G 1L7, Canada; Princess Margaret Cancer Centre, University Health Network, 610 University Avenue, Toronto, ON M5G 2M9, Canada; Broad Institute of MIT and Harvard, 415 Main Street, Cambridge, Cambridge, MA 02142, United States; Broad Institute of MIT and Harvard, 415 Main Street, Cambridge, Cambridge, MA 02142, United States; Department of Medical Oncology, Dana-Farber Cancer Institute, 450 Brookline Avenue, Boston, MA 02115, United States; Department of Medical Oncology, Dana-Farber Cancer Institute, 450 Brookline Avenue, Boston, MA 02115, United States; Department of Medical Oncology, Dana-Farber Cancer Institute, 450 Brookline Avenue, Boston, MA 02115, United States; Department of Medical Oncology, Dana-Farber Cancer Institute, 450 Brookline Avenue, Boston, MA 02115, United States; Broad Institute of MIT and Harvard, 415 Main Street, Cambridge, Cambridge, MA 02142, United States; Harvard Medical School, 25 Shattuck Street, Boston, MA 02115, United States; Broad Institute of MIT and Harvard, 415 Main Street, Cambridge, Cambridge, MA 02142, United States; Department of Medical Oncology, Dana-Farber Cancer Institute, 450 Brookline Avenue, Boston, MA 02115, United States; Hematology Branch, National Heart, Lung, and Blood Institute, National Institutes of Health, 10 Center Drive, Bethesda, MD 20892, United States; Hematology Branch, National Heart, Lung, and Blood Institute, National Institutes of Health, 10 Center Drive, Bethesda, MD 20892, United States; Department of Medical Biophysics, University of Toronto, 101 College Street, Toronto, ON M5G 1L7, Canada; Princess Margaret Cancer Centre, University Health Network, 610 University Avenue, Toronto, ON M5G 2M9, Canada; Ontario Institute for Cancer Research, 661 University Avenue, Suite 510, Toronto, ON M5G 0A3, Canada; Harvard Medical School, 25 Shattuck Street, Boston, MA 02115, United States; Broad Institute of MIT and Harvard, 415 Main Street, Cambridge, Cambridge, MA 02142, United States; Department of Medical Oncology, Dana-Farber Cancer Institute, 450 Brookline Avenue, Boston, MA 02115, United States; Harvard Medical School, 25 Shattuck Street, Boston, MA 02115, United States; Broad Institute of MIT and Harvard, 415 Main Street, Cambridge, Cambridge, MA 02142, United States; Department of Medical Oncology, Dana-Farber Cancer Institute, 450 Brookline Avenue, Boston, MA 02115, United States; Harvard Medical School, 25 Shattuck Street, Boston, MA 02115, United States; Broad Institute of MIT and Harvard, 415 Main Street, Cambridge, Cambridge, MA 02142, United States; Division of Hematology/Oncology, Boston Children’s Hospital, 300 Longwood Avenue, Boston, MA 02115, United States; Howard Hughes Medical Institute, 20 Shattuck St, Boston, MA 02115, United States; Harvard Medical School, 25 Shattuck Street, Boston, MA 02115, United States; Broad Institute of MIT and Harvard, 415 Main Street, Cambridge, Cambridge, MA 02142, United States; Krantz Family Center for Cancer Research and Department of Pathology, Massachusetts General Hospital, 55 Fruit Street, Boston, MA 02114, United States

**Keywords:** CNV inference, single-cell multiome, cancer evolution

## Abstract

Aberrant alterations in genome copy number, chromatin accessibility, and transcriptional programs all play pivotal roles in cancer. The Numbat algorithm has been widely adopted to perform copy number variation (CNV) inference from single-cell RNA sequencing (scRNA-seq) data. Here, we introduce Numbat-multiome that extends the capabilities of Numbat to perform CNV inference from both scRNA-seq and accessible chromatin profiles (through single-cell Assay of Transposase [Tn5]-Accessible Chromatin sequencing [scATAC-seq] data), either separately or in an integrated manner. Our approach unifies data originating from different modalities through a binning strategy that relies on a common genomic coordinate system across modalities. We demonstrate the tool’s robust performance in four running modes (RNA gene, RNA bin, ATAC bin, and Combined bin) using benchmark cohorts of tumors with dynamic changes in expression patterns and copy number heterogeneity, including early-stage multiple myeloma and Richter’s syndrome arising from chronic lymphocytic leukemia, validated against whole-genome sequencing. Numbat-multiome achieves high precision and recall (median F1>0.9) across different CNV event types, with consistent performance across sample types and event lengths. The tool’s ability to track clonal evolution in serial samples and identify rare subclones allows for integration of epigenomic profiles at the subclonal level, providing new insights into the stepwise genetic and epigenetic changes underlying cancer phenotypic shifts.

## Introduction

Intratumor heterogeneity arises from both genetic and epigenetic heritable changes that accumulate in cells and shape the dynamics of their phenotypes, which can ultimately lead to progression and transformation in cancer [1]. Single-cell (sc) methods capture these changes reflected in diverse modalities: single-cell RNA sequencing (scRNA-seq) quantifies transcript levels [2, 3], while single-cell Assay of Transposase (Tn5)-Accessible Chromatin sequencing (scATAC-seq) detects regions of accessible chromatin [4] where transcriptional machinery can bind and take action as a complex to initiate transcription. scATAC-seq data provide signals to construct regulatory units that control the transcription levels, which are then measured by scRNA-seq. This includes regulatory elements (e.g. promoters and enhancers), regulators (e.g. transcription factors [TFs] and cofactors), and their target genes. Previous efforts have focused on integrating scRNA- and scATAC-seq data based on presumed relationships between accessible regulatory elements and gene expression patterns, especially when these two types of data are not obtained from the same group of cells [5, 6]. However, cell lineage information has not been widely used as the anchor to align these two types of data.

Natural lineage markers, such as copy number variations (CNVs), hold strong potential for dissecting clonal evolution [7–10]. Large-scale somatic CNVs can be robustly detected in both scRNA- and scATAC-seq data; moreover, in many cases, CNVs continue to accumulate with tumor evolution and thus can be used to construct the tumor clonal architecture [9–11]. Despite this potential for dissecting clonal evolution from inferred CNV events, uneven coverage and higher sparsity in typical scATAC-seq data relative to scRNA-seq data introduce challenges, especially due to lower signal-to-noise ratios to distinguish CNVs and noisier haplotype phasing [10]. Existing methods partly address these issues but can require additional experimental inputs such as coupled scDNA-seq data [10] or extensive parameter learning as proposed in the binary segmentation algorithm [11]. Building on Numbat [9], a widely adopted iterative framework that infers CNVs and reconstructs subclonal phylogenies primarily from scRNA-seq data, we present Numbat-multiome: an extended pipeline designed to handle scATAC-seq data alone or in combination with paired scRNA-seq data. By unifying the signals from differential molecular abundance (i.e. relative total coverage), allelic imbalance denoised by population-based haplotype phasing across both modalities, Numbat-multiome leverages a unique segmentation strategy that significantly enhances the detection of copy-neutral loss-of-heterozygosity (CNLoH) events that cannot be identified by coverage-based methods alone. Consequently, Numbat-multiome robustly infers both segmentation and per-clone copy-number estimates for each genomic segment. Numbat-multiome enables researchers with only scATAC-seq data, or with paired scRNA-seq and scATAC-seq datasets, to identify major CNV events and infer clonal structures, without requiring whole-exome or whole-genome sequencing (WGS) data.

Here, we benchmarked Numbat-multiome in two scenarios with clear changes in cellular phenotypes often accompanied with CNVs during disease progression: (i) natural evolution of precursor stages (smoldering multiple myeloma [SMM]) preceding the development of overt malignant multiple myeloma (MM), a slow-growing cancer that accumulates CNVs; and (ii) a case of Richter’s syndrome (RS), which is a dynamic transformation from one malignancy, chronic lymphocytic leukemia (CLL), to a more aggressive lymphoma (most common histology, diffuse large B-cell lymphoma [DLBCL]).

## Materials and methods

### Sample collections and sequencing in both cohorts

#### Sample collection

Bone marrow (BM) aspirates were collected from donors/patients according to the declaration of Helsinki and processed immediately to ensure high-quality single-cell suspensions. The aspirates were first passed through a 40 $\mu $m cell strainer to remove debris and clumps. Red blood cells were lysed using Ammonium-Chloride-Potassium lysis buffer for 5 min at room temperature, followed by quenching with phosphate-buffered saline (PBS) containing 0.04% bovine serum albumin (BSA). Cells were then centrifuged at 500 $\times $g (times gravity) for 5 min, resuspended in PBS + 0.04% BSA, and counted using a hemocytometer or an automated cell counter.

MM samples were collected under IRB no. 14-174 from D ana-Farber/Harvard Cancer Center (DF/HCC). Magnetic beads selection (with autoMACS) was performed to enrich plasma cells in the collected sample, as plasma cells are in much lower abundance in the original BM samples.

RS and CLL samples were collected from a patient enrolled in a phase 2 study of acalabrutinib for high-risk CLL (ClinicalTrials.gov ID NCT02337829). The study was approved by the National Heart, Lung, and Blood Institute Institutional Review Board. Blood and marrow mononuclear cells were isolated by density gradient centrifugation and cryopreserved until time of single-cell assay preparation.

Other than the sample (MM3_T2) collected at the later time point from MM3, all samples have scRNA-seq, scATAC-seq, and WGS data profiled from the same specimen collection.

#### Sample processing for scRNA-seq

An aliquot of $\sim $50,000 cells was taken and resuspended in PBS + 0.04% BSA before being immediately processed using the 10$\times $ Genomics Chromium platform following the manufacturer’s protocol for the Single Cell 5’ v3.1 kit (MM samples) and 5’ HT kit v2 (CLL/RS samples). Cell viability (>85%) was ensured using trypan blue staining before loading onto the Chromium Controller instrument (10$\times $ Genomics).

#### Sample processing for scATAC-seq

A separate aliquot of $\sim $50,000 cells was processed following the 10$\times $ Genomics Chromium Single Cell ATAC v2 protocol. Nuclei were isolated using lysis buffer (10 mM Tris–HCl, 10 mM NaCl, 3 mM Mg$\text{Cl}_{2}$, 0.1% NP-40, 0.1% Tween-20, and 0.01% Digitonin), incubated on ice for 3 min, and washed with resuspension buffer (PBS + 0.04% BSA). The nuclei were counted and adjusted to the required concentration before transposition with Tn5 transposase. For CLL/RS samples, this was done as previously reported [12] using the 10$\times $ Genomics Chromium Single Cell ATAC v2 kit enriched for mitochondria genomic reads.

#### Library preparation for scRNA- and scATAC-seq

Both libraries were prepared according to the respective 10$\times $ Genomics workflows and sequenced on an Illumina NovaSeq 6000 (for MM samples), or a NovaSeq X flowcell SP (for CLL/RS samples), with a target read depth of $\sim $50,000 reads per cell for scRNA-seq, and $\sim $25,000 reads per nucleus for scATAC-seq.

#### Library preparation and sequencing for whole-genome sequencing

The DNA library preparation and WGS of (S)MM patient samples have been previously described by Alberge *et al*. [13]. In brief, DNA was extracted using the Monarch Genomic DNA Purification Kit and quantified with a Qubit 3.0 fluorometer. Libraries were constructed using the NEBNext Ultra II FS kit with unique dual indices, and fragment size and concentration were assessed using a BioAnalyzer and qPCR. Final pooled libraries underwent $2\times 150$ bp paired-end WGS on the Illumina NovaSeq6000 platform (S4 flowcells) at the Broad Institute Genomics Platform.

For CLL/RS samples, DNA extraction (Qiagen, Cat no.51304) was performed on $1\times 10^{6}$ cells from the live fraction of either total cells or CD3-depleted (Biolegend, Cat no. 480134) samples for WGS of the malignant B-cell “tumor” population so as to preserve $ \geqq $70% tumor purity.

### scRNA- and scATAC-seq data processing

We performed initial read quality control (QC) and preprocessing, from raw Illumina bcl signals, to reads aligned to GRCh38 human reference genome for scRNA- and scATAC-seq data using CellRanger RNA (10$\times $ Genomics, version 8.0.0) [2] and CellRanger ATAC (10$\times $ Genomics, version 2.0.0) [14]. Downstream QC steps (e.g. low-quality cells and doublets [15]), dimension reduction, cell clustering, and initial cell type annotation for scRNA- and scATAC-seq were conducted with Scanpy [16] and pyCistopic [17], respectively. Detailed implementation and parameter choices can be found in supplementary methods.

### Integration of scRNA-seq and scATAC-seq data using SCENIC+

To comprehensively map gene regulatory networks (GRNs) during clonal evolution, we leveraged the SCENIC+ computational framework [17], designed to identify enhancer-driven regulons (eRegulons). SCENIC+ integrates TF expression, chromatin accessibility, and motif enrichment to reconstruct enhancer-based GRNs at single-cell resolution.

#### Pseudo-multiome metacell generation

Given that our scRNA- and scATAC-seq datasets originated from separate cell populations, we integrated these datasets into pseudo-multiome datasets following the SCENIC+ protocol. Briefly, we randomly sampled and averaged counts from 50 cells (the default) per CNV-defined subclone from each modality, generating representative metacells containing paired transcriptional and chromatin accessibility data. The number of metacells per subclone was automatically determined to avoid oversampling individual cells (maximally sampling twice).

#### Candidate enhancer and motif enrichment identification

Candidate enhancers were identified using pycisTopic [18], clustering accessible regions into co-accessible topics. SCENIC+ utilized these topics alongside differentially accessible regions as candidate enhancers. Motif enrichment within these regions was assessed using pycisTarget, employing cisTarget ranking-and-recovery and differential enrichment of motif methods, supported by a curated motif database (32,765 motifs for >1500 TFs).

#### Quantification of regulatory relationships

We quantified TF-to-gene and region-to-gene relationships using Arboreto (GRNBoost2 [19]). TF-to-gene scores predicted TF expression from gene expression counts, while region-to-gene scores were calculated from chromatin accessibility around each gene ($\pm $1–150 kb, a minimum of 1 kb and a maximum of 150 kb upstream/downstream of the start/end of the gene). Pearson and Spearman correlations distinguished positive from negative regulatory interactions, respectively.

#### Construction and scoring of eRegulons

eRegulons (enhancer regulons) were constructed linking TFs to candidate enhancer regions and target genes. SCENIC+ identifies two eRegulon classes: direct (motifs with direct TF evidence) and extended (motifs with indirect, orthology-based evidence). Direct eRegulons were prioritized; extended eRegulons were retained only when direct evidence was absent. Significant TF-target gene pairs (minimum 10 target genes) were identified via gene set enrichment analysis. eRegulon activity was scored per metacell using AUCell [20], generating regulatory state matrices.

#### Downstream analysis and visualization

The eRegulon activity matrix was subjected to dimensionality reduction using Uniform Manifold Approximation and Projection (UMAP) [21], enabling visualization and clustering based on regulatory profiles. This approach elucidated dynamic regulatory states linked to clonal evolution and transformation from CLL to RS.

### Binning strategy for both scRNA-seq and scATAC-seq

To decide on the common feature space to unify the scRNA- and scATAC-seq data for interring CNVs, we followed prior publications and used a set of bins with an average size of 200 kb [9–11, 22]. The basic rationale behind this choice of bins was to capture at least one expression unit per bin, consisting of a gene body and its surrounding regulatory genomic regions. Theoretically, when transcription is active, mRNA molecules and Tn5 accessible chromatin fragments in such expression units will have proportional abundance, the same heterozygous single-nucleotide polymorphism (SNP) readouts, and a comparable allele-specific capture rate. Thus, any CNV covering these expression units could be reliably inferred from both the scRNA- and scATAC-seq data. We obtained this set of bins from the CopyKit package hg38_grangeslist  internal data sets. A series of bin sizes have been explored by Gao *et al*. [22] and implemented in the CopyKAT package. This bin set is the default in Numbat-multiome, but the user can supply a different bin set, if desired.

### Aggregating counts in bins for the RNA bin, ATAC bin, and combined bin running modes

After performing a basic preprocessing and QC check with Cell Ranger (10× Genomics) implementations (or other similar tools), the most common starting data formats are cell-by-gene matrices for scRNA-seq and fragment count files across cells for scATAC-seq (or cell-by-peak calling as a more denoised version of scATAC-seq data). We provided customized scripts to perform the conversion from: (i) cell-by-gene matrix to cell-by-bin matrix used in the RNA bin mode; (ii) fragment count across cells to cell-by-bin matrix for mode ATAC bin (recommended); (iii) cell-by-peak matrix to cell-by-bin matrix (for mode ATAC bin). The general idea behind all conversions is similar: find genes/peaks/fragments falling within the genomic coordinates of each bin (findOverlaps() in GenomicRanges package). The original count data of genes/peaks/fragments were summed up to compute the count data in bins. The resulting RNA bin and ATAC bin count matrices were concatenated vertically, sharing the same bin columns.

### Reference count data generation

To detect the differential count abundance, the choice and preparation of the count reference matter. As a natural extension to the original Numbat implementation, we used the bin-by-[cell type] matrix of normalized count values when running with modes RNA bin and ATAC bin instead of gene-by-[cell type] matrix of normalized expression values (raw feature counts divided by total counts). The original function aggregate_counts() in Numbat was used but with the supply of raw bin-by-normal cells count matrix as the count_mat parameter. Normal plasma cells were subsampled ($N=1500$, $\text{seed}=123$) from five healthy donors in the case of MM. In the case of CLL-to-RS, we used both normal B cells and other detected immune cells (T cells, monocytes, and erythrocytes) to form the count reference (maximum cells allowed per cell type was 1500, seed was set to 123 when subsampling was required). The cell type annotation in both scRNA- and scATAC-seq data was based on well-established marker genes (Supplementary methods).

Combined bin mode requires the supply of horizontally combined reference of RNA bin and ATAC bin to allow the optimal reference match when running the core Numbat algorithm.

### Genotyping and phasing in the combined bin mode

Accurate detection of CNVs by Numbat-multiome strongly relies on leveraging allelic imbalance information, necessitating robust genotyping and phasing across both scRNA-seq and scATAC-seq modalities. While the original Numbat genotyping script (pileup_and_phase.R) functions effectively for individual RNA or ATAC bin modes using a single --UMItag, it lacks the capability for simultaneous processing required by the combined bin mode.

To address this limitation, we adapted pileup_and_phase.R to accept multiple --UMItag values as a vector, enabling simultaneous allele counting from both RNA- and ATAC-derived aligned BAM files. This modification ensures consistent and integrated genotyping and phasing across both modalities. Subsequently, allele counts generated from these datasets are jointly phased using Eagle2 [23] with reference haplotypes from the 1000 Genomes Project Consortium [24].

In brief, Eagle2 leverages known population haplotype frequencies, computationally inferring individual variant haplotypes and addressing potential phase-switch errors through an inhomogeneous Markov model, wherein site-specific phase-switch probabilities scale exponentially with inter-SNP distances. This approach effectively mitigates noise introduced by allele-specific transcriptional variability, improving the CNV inference CNV inference accuracy. Thus, our adapted genotyping and phasing pipeline ensures robust, modality-integrated data preparation, critical for downstream CNV inference in Numbat-multiome’s combined bin mode (Supplementary Fig. S6).

### Connection to Numbat core inference algorithm

The core Numbat inference function can be seamlessly used with the prepared inputs as described above with a minor change to the main gtf file. Briefly, the original gtf data contains the coordinates of genes and serve as the link between SNP allele count and gene count data, bridging the combination of signals from these two sources of information. Since these coordinates are tightly linked to the input genome_version, the function is hard-coded to use one of the internal gtf data based on the specified genome version. However, since the feature space in our proposed three modes is defined by bins rather than genes, we need an equivalent data object to build the linkage between SNPs and bins. Such information has already been used when we prepare the aggregated count data per bin and should be readily available for running the core inference. We therefore changed the main inference function of Numbat to enable a user-defined gtf input.

Numbat-multiome integrates allelic imbalance into CNV inference using the core methodology established by Numbat [9]. Specifically, it employs two complementary signals within a joint hidden Markov model (HMM): coverage counts (originally gene expression counts in Numbat, now genomic bin counts in Numbat-multiome), and phased haplotype allele frequencies.

Coverage counts per bin are modeled via a Poisson Log-Normal distribution, which accounts for variability in chromosomal dosage, library size, and baseline expression or coverage variability. Phased paternal (chosen arbitrarily) haplotype frequencies are modeled with a Beta-Binomial distribution to capture allelic imbalance signals, utilizing robust population-based phasing (with Eagle2) to enhance sensitivity. These signals are integrated within an expanded-state HMM, containing 15 discrete states that characterize joint chromosomal dosage and haplotype fraction scenarios, including neutral, amplification (Amp), deletion (Del) and CNLoH.

By simultaneously leveraging bin-count coverage and allelic imbalance, Numbat-multiome achieves enhanced accuracy in detecting and characterizing complex genomic aberrations, especially events like CNLoH, at single-cell resolution.

### Whole-genome sequencing copy number variation inference with AllelicCapSeg

Because WGS segmentation results are treated as the ground truth throughout this work, both their accuracy and sensitivity are important. We used a widely tested algorithm AllelicCapSeg [13, 25] to identify and quantify allele-specific somatic CNVs. In brief, AllelicCapSeg analyzes genomic data to identify regions with altered copy number and, for each region, calculates the allelic imbalance between the two alleles based on read counts of both alleles in germline heterozygous SNPs. ABSOLUTE then uses these estimated allele-specific copy number ratios to infer the proportion of tumor cells in the sample (purity) and the overall amount of DNA per tumor cell (ploidy), as well as rescaling the copy-number estimates to reflect only the cancer cells (by removing the normal counterpart’s contribution). We used the coordinates of segments as well as their inferred total copy numbers from AllelicCapSeg for evaluating the CNV inferences of Numbat-multiome.

### Precision, recall, and F1 calculations for segmentation consistency

When treating WGS segmentation as the ground truth, we sought out to evaluate the accuracy and sensitivity of the CNV inference using the different modes of Numbat-multiome. We used the scaled copy-number estimates from AllelicCapSeg and ABSOLUTE to infer copy-number alterations using the thresholds of <1.3 for Dels and >2.5 for Amps. By comparing these ground-truth results to the genomic coordinates and copy-number states returned by Numbat-multiome, we can calculate precision and recall in the following way:


\begin{align*} &\mathrm{Precision} = \frac{\text{Overlapped CNV Segment Length}}{\text{Total CNV Segment Length from Numbat-multiome}}\end{align*}

\begin{align*} &\mathrm{Recall} = \frac{\text{Overlapped CNV Segment Length}}{\text{Total CNV Segment Length from AllelicCapSeg}}\end{align*}

\begin{align*} &\mathrm{F1} = \frac{2\times \mathrm{Precision}\times\mathrm{Recall}}{\mathrm{Precision}+\mathrm{Recall}}\end{align*}

The same calculation of precision and recall were used when we stratified CNVs events based on their status or arm-level vs. focal events in the following two ways:

1. We first separated CNV segments by their Numbat-multiome inferred status: (i) Amp, (ii) balanced Amplification (bAmp): both alleles are amplified while maintaining their allelic balance, (iii) Del, and (iv) CNLoH: no change in total copy number, but zero copies of the minor allele.
2. We then combined segments for each arm-level vs. focal CNV events, as described below.

### Arm/focal and cytogenetic annotations of copy number variation events

Due to the bias in DNA amplification and capture methods, the coverage (even with WGS) is not completely uniform. Moreover, with technologies like 10$\times $ scRNA-seq and scATAC-seq, the dropout and inherent selectivity in the genome-wide profiled molecules tend to yield over-segmented copy-number profiles. Therefore, when aiming to quantify the inference performance at the level of individual CNV events, we first annotate the chromosome arm of each event and then combine neighboring segments. We use the two Numbat files (centromere regions:acen_hg38 and chromosome sizes: chrom_sizes_hg38) to assign a chromosome arm (p or q) for each event, while excluding the last 10 kb of each chromosome arm to avoid telomeres (centromeres are already removed in the files). We combine consecutive segments in the same arm, if they have the same status (Amp, bAmp, Del, or CNLoH) and together span >80% of the chromosome arm (e.g. Chr5p-arm-Amplification). In the case of consecutive segment(s) with CNV status covering >10% but <80% of the chromosome arm, we annotate those segments as a focal arm event with further detailed cytogenetic band information (based on the cytogene annotation downloaded from UCSC Genomics Institute’s Genomic Browser). We did not consider segments covering <10% of the arm or <200 kb in length.

### Benchmarking with other copy number variation inference tools

We conducted benchmarking analyses using two publicly available methods: epiAneufinder [11] and CONGAS+ [26].

Briefly, epiAneufinder is based on a fixed genomic binning approach, by default using bins of 100 kb. The method infers CNV states by comparing read depth within bins to a genome-wide average, categorizing segments into discrete copy number states (loss, normal, or gain). This is achieved by segmenting the genome using Anderson–Darling distances, identifying likely copy number breakpoints, and assigning segments to CNA states based on mean read coverage. Cells are subsequently clustered based on the similarity of their gain and loss profiles to identify clonal heterogeneity. Notably, epiAneufinder does not utilize allelic information and lacks the ability to perform a unified genomic segmentation across cells.

In short, CONGAS+ employs a Bayesian probabilistic framework integrating both scRNA-seq and scATAC-seq data. It models read counts from each modality using a Negative Binomial distribution. The CONGAS+ algorithm assigns the probability for each CNV using a Dirichlet prior distribution. By default, if the expected ploidy is $p$, the Dirichlet vector assigns a value of 0.6 to the $p$th entry and 0.1 to the remaining entries to skew the distribution toward the anticipated copy number state [0,1,3,4]. For this benchmarking, we set the expected ploidy as 2, assuming a diploid genome (ploidy=2) for all genomic segments.

To ensure fair comparisons, the same set of cells were used across all methods, and low-coverage bins (not found in selected cells) were uniformly removed prior to analysis in CONGAS+, epiAneufinder, and Numbat-multiome.

Numbat-multiome was run in two distinct modes: the ATAC bin mode (using only scATAC-seq data) and the Combined bin mode (integrating scRNA-seq and scATAC-seq data). We evaluated performance metrics, including precision, recall, and F1-score, by comparing inferred CNV profiles from each method against ground-truth CNVs derived from matched WGS data.

All analyses and benchmarking metrics were computed using custom scripts developed for this study. These scripts and associated parameters are publicly available at https://github.com/getzlab/Numbat-multiome_Analysis.

### Bin size evaluation for copy number variation detection

To evaluate the trade-off between bin size, CNV detection resolution, and computational cost, we systematically tested a series of bin sizes (500 kb, 200 kb, 100 kb, and 50 kb) on a dataset comprising 2500 cells, analyzed on an 8-core machine with 16 GB RAM. The sample chosen for analysis was characterized by known chromothripsis events on chromosome 7, facilitating the assessment of breakpoint detection capabilities across varying resolutions.

CNV detection results were visually compared to assess breakpoint granularity and the identification of focal events at each bin size. Computational runtime was systematically measured and plotted against bin sizes to quantify computational costs, demonstrating scalability and efficiency trade-offs across resolutions.

All analyses were executed using custom scripts specifically developed for this evaluation, available in the supplementary materials.

## Results and discussion

### Numbat-multiome can be adapted to include scATAC-seq data, and has four running modes

Studying the dynamics of regulatory units can reveal candidate regulators of gene programs associated with clonal evolution in cancer. The inference of clonal evolution based on CNV events from scRNA- and scATAC-seq data remains a key challenge in studying gene regulation and dynamics. While Numbat has been widely adopted to accurately infer CNV events from scRNA-seq data, a comparable method utilizing population phasing to conduct haplotype-aware CNV inference does not yet exist for scATAC-seq data, and in particular for the case of sample-paired scRNA-seq and scATAC-seq (i.e. when scRNA- and scATAC-seq data are generated from different cells from the same sample).

The main rationale behind Numbat-multiome is to project the genomic coverage information from scATAC- and scRNA-seq data in the same space and then jointly process the two modalities using the original core Numbat inference algorithm. Differential molecule abundance and haplotype-enhanced allelic imbalance are the two main signals Numbat uses in its iterative framework for inferring CNVs from scRNA-seq data [9]. We rationalized that similar information could be extracted from scRNA- and scATAC-seq data. Since heterozygous SNPs are present and can be detected throughout the genome, including in the coding and non-coding regions, both scATAC- and scRNA-seq data can be used to measure allelic imbalance. In fact, since scATAC-seq data cover a wider range of regulatory genomic regions, which include a larger number of SNPs relative to scRNA-seq data, it can provide additional information to estimate allelic imbalance across the genome.

**Figure 1:**
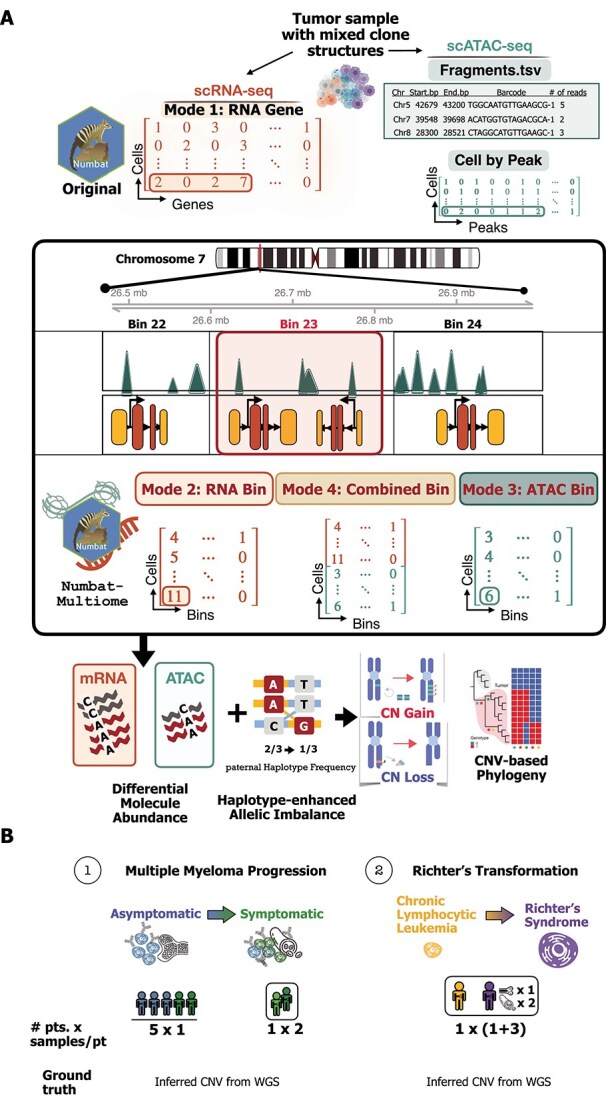
Versatile running modes of Numbat-multiome enable robust CNV inference from paired scRNA-seq and scATAC-seq data. (A) Schematic illustrating the four distinct running modes of Numbat-multiome, including the original RNA gene mode from Numbat and three additional modes: RNA bin, ATAC bin, and a Combined (RNA+ATAC) bin mode. (B) Benchmarking framework used to evaluate the performance of Numbat-multiome using two cohorts: an early-stage multiple myeloma set (MM1–3, SMM1–3) and a CLL-to-RS case (Patient A) with four tumor samples collected serially over time (one collected during the CLL phase of their disease, three collected after the CLL transformed to RS).

However, unlike the availability of using transcripts (genes) to quantify molecular abundance in scRNA-seq data, there is no comparable universal feature space defined for quantifying molecular abundance in scATAC-seq data. Inspired by the binning strategy adopted by previous tools for CNV inference with scATAC-seq [10, 11], we collapsed fragments or peak counts falling into a user-supplied, genome-wide bin set to generate a cell-by-bin count matrix. We typically use bins of size $\sim $200 kb (Materials and methods). In the case of sample-paired multiome, we reaggregated the cell-by-gene count matrix from scRNA-seq data to be compatible with the cell-by-bin matrix from scATAC-seq data by summing read counts from genes that fall into the same bin. A vertical concatenation of the two cell-by-bin matrices derived from scRNA- and scATAC-seq data results in a combined bin matrix ready for a joint Numbat CNV inference (Fig. 1A).

To increase the flexibility of the Numbat-multiome pipeline, we designed the tool to run in four modes: (i) Mode 1 corresponds to the original Numbat algorithm, referred to as RNA gene; (ii) Mode 2 uses the cell-by-bin matrix derived from scRNA-seq data, referred to as RNA bin; (iii) Mode 3 uses the cell-by-bin matrix derived from scATAC-seq data, referred to as ATAC bin; and (iv) Mode 4 uses the vertically combined cell-by-bin matrix for sample-paired multiome, referred to as Combined bin (Fig. 1A).

We benchmarked the four modes of Numbat-multiome in tumor samples for which dynamic changes occur during disease progression, including seven samples collected from patients at different stages of MM (from precursors to overt myeloma) and four serial samples collected from a CLL patient before and after transformation to RS (Fig. 1B). In both tumor types, we used a case in which serial samples were collected over time from the same patient to confirm that the inferred clonal evolution matches the tumor chronology. In all cases but MM3_T2, we generated three data modalities (WGS, scRNA-, and scATAC-seq data) for each sample. To evaluate the performance of detecting clonal and subclonal CNV events, we assume that three data modalities share the same clonal composition (even though they were generated on different cells from the same sample). Since WGS has the most even and consistent genomic coverage and is commonly used to infer CNVs, we used the WGS-based CNVs (estimated using both total coverage and allelic imbalance) as the ground truth (Materials and methods).

### Robust performance found across running modes and samples

To evaluate the performance of Numbat-multiome, we applied all four Numbat-multiome modes to eight datasets (for a total of 32 Numbat-multiome runs [see Materials and methods]): seven single-tumor samples from six patients with smoldering multiple myeloma or multiple myeloma (one dataset per sample; Fig. 1B), and one aggregated dataset combining four serial samples from a single patient spanning their disease course from CLL to RS.

**Figure 2:**
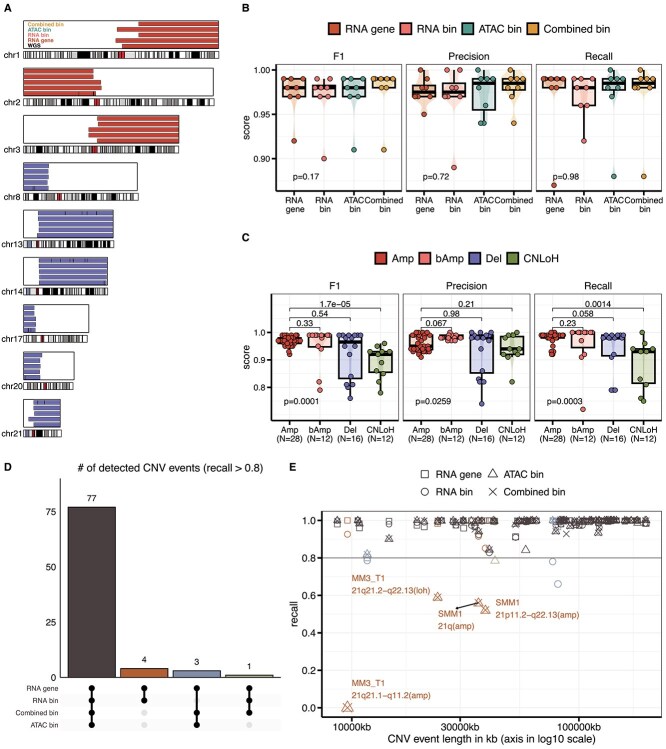
Numbat-multiome robustly inferred CNVs using all four modes compared to WGS data. (A) CNV segmentation profiles for multiple myeloma patient sample MM1 are shown for select chromosomes with color representing amplification (Amp) or deletion (Del), and tracks ordered bottom to top: WGS (AllelicCapSeg), RNA gene, RNA bin, ATAC bin, and Combined bin. (B) Violin plots with embedded boxplots and data points summarize per-sample precision, recall, and F1-score ($N=8$), with comparison of linear mixed models treating sample ID as a random effect, annotated in each panel’s bottom-left. (C) CNV event types (Amp, bAmp, Del, CNLoH) across runs (Materials and methods) were annotated from Numbat’s 15-state hidden Markov model (HMM) using total dosage ($\phi $) and paternal haplotype fraction ($\theta $); labels report significance values (p-values) from linear mixed models and Wilcoxon rank-sum tests that were run with Amp as the reference level. (D) An UpSet plot summarizes detection of all CNV events ($N=86$) across modes, with the combination matrix indicating mode intersections, where the number on top of the bars give the jointly detected event counts per combination, and where detection is defined as recall $\geq 0.8$. (E) A scatterplot maps CNV length to recall, grouping points by the detection subsets defined in (D) and labeling events with recall <0.6 by sample, CNV annotation, and mode.

By aggregating all inferred CNV events, we first evaluated the performance of Numbat-multiome in detecting nondiploid regions in the genome identified using AllelicCapSeg applied to the paired WGS data (Fig. 2A, Materials and methods). At the sample level, all Numbat-multiome runs achieved high inference performance when measured with precision, recall, and derived F1 (median F1s > 0.9; Fig. 2B, Supplementary Table 1, Materials and methods). No significant associations between running modes and three different metrics were found using linear mixed models ($P$-values =0.17, 0.72, and 0.98 for F1, precision, and recall, respectively, Fig. 2B). Most of the variations in the results across running modes arose from minor differences in the inferred genomic coordinates of breakpoints (as shown in the case of MM1: Fig. 2A).

We confirmed the high performance across different types of CNV events (unbalanced amplifications [Amps] and balanced amplifications [bAmps]; deletions [Dels]; and CNLoH; Fig. 2C). Notably, we observed a lower recall (Fig. 2C–E) in some cases of bAmps, Dels, and CNLoHs.

We detected 77 out of 86 CNV events (59 Amps, 5 bAmps, 15 Dels, and 7 CNLoHs) across the full benchmark cohort, with recall >0.8 in all four modes (Fig. 2D; Supplementary Fig. S1). On average, each sample had $\sim $10 CNV events detected (Supplementary Fig. S1). Other than one event detected with low recall by all four Numbat-multiome modes (a 14q focal Del in sample SMM3), all events could be detected with at least two running modes (Fig. 2D). Because $\sim $200 kb has been previously implicated [22] as the average genome length containing at least one expression unit (Materials and methods), we followed Gao *et al*. [22] and chose the 220-kb variable genomic bin set as our default bin set in Numbat-multiome. We did, however, encounter some challenges of identifying focal CNVs when using this bin size, as reflected by lower recall in several cases of 21q CNV events (Fig. 2E; Supplementary Fig. S4). We recommend optimizing the bins based on the density of available data and desired resolution for detecting CNV events, taking into account that a larger number of bins can increase the noise and run time.

### Benchmarking Numbat-multiome against other copy number variation inference tools

To compare the performance of Numbat-multiome to other existing CNV inference tools, we chose to benchmark it against two established methods: epiAneufinder [11] (scATAC-seq data only) and CONGAS+ [26] (jointly with scRNA-seq and scATAC-seq data). We assessed each tool using ground-truth WGS data, examining precision, recall, and overall accuracy measured by the F1-score.

In comparisons against epiAneufinder, Numbat-multiome’s ATAC bin mode demonstrated superior accuracy, particularly in the identification of Del events (Supplementary Fig. S2). epiAneufinder exhibited fragmented and less consistent CNV calls due to (i) the lack of use of allelic imbalance, (ii) not performing segmentation on a cell-by-cell basis, and (iii) not obtaining a consensus segmentation across the entire cell population.

When compared against CONGAS+, Numbat-multiome’s combined bin mode significantly outperformed it in all evaluation metrics (Supplementary Fig. S2). The limited performance of CONGAS+ likely arises from its reliance on prior knowledge of genomic segments and their ploidy; in our unbiased benchmarking setting, where no prior CNV knowledge was provided, CONGAS+ was less accurate in inferring complex CNV landscapes.

These results confirm that Numbat-multiome achieves highly accurate CNV inference compared to other existing tools. The benefits of Numbat-multiome are driven by its integrated modeling of both molecule abundance and haplotype-informed allelic imbalance across cell populations.

### Impact of bin sizes on resolution of focal copy number variation detection and computational cost

To systematically evaluate the influence of the bin size on the resolution of detected CNVs and the computational cost, we analyzed our CLL-to-RS case known to exhibit chromothripsis on chromosome 7. This biological context allowed us to benchmark the granularity of detected CNV breakpoints at varying bin sizes.

As illustrated in Supplementary Fig. S3a, reducing the bin size from 500 kb down to 50 kb markedly improved CNV breakpoint resolution. Smaller bins revealed numerous additional CNV transitions, particularly in regions of known structural complexity associated with chromothripsis. Specifically, bin sizes of 50–100 kb allowed clearer detection of focal events, demonstrating enhanced sensitivity and resolution for fine-scale genomic alterations.

However, improved resolution at smaller bin sizes also introduced higher segmentation noise and a significant increase in the computational time. Supplementary Fig. S3b demonstrates the computational burden scaling nearly log-linearly with decreasing bin sizes. Notably, runtime increased by more than four-fold when decreasing bin sizes from 500 kb to 50 kb, highlighting the increased computational burden associated with finer segmentation.

Considering these findings, we recommend using by default a 200-kb bin size for typical analyses, balancing computational efficiency with sufficient resolution. Nonetheless, we recognize that there are scenarios, such as clinical CNV diagnostics or detailed chromothripsis, that may justify using smaller bin sizes (Materials and methods).

### Detection of clonal evolution over time using serially collected samples

**Figure 3:**
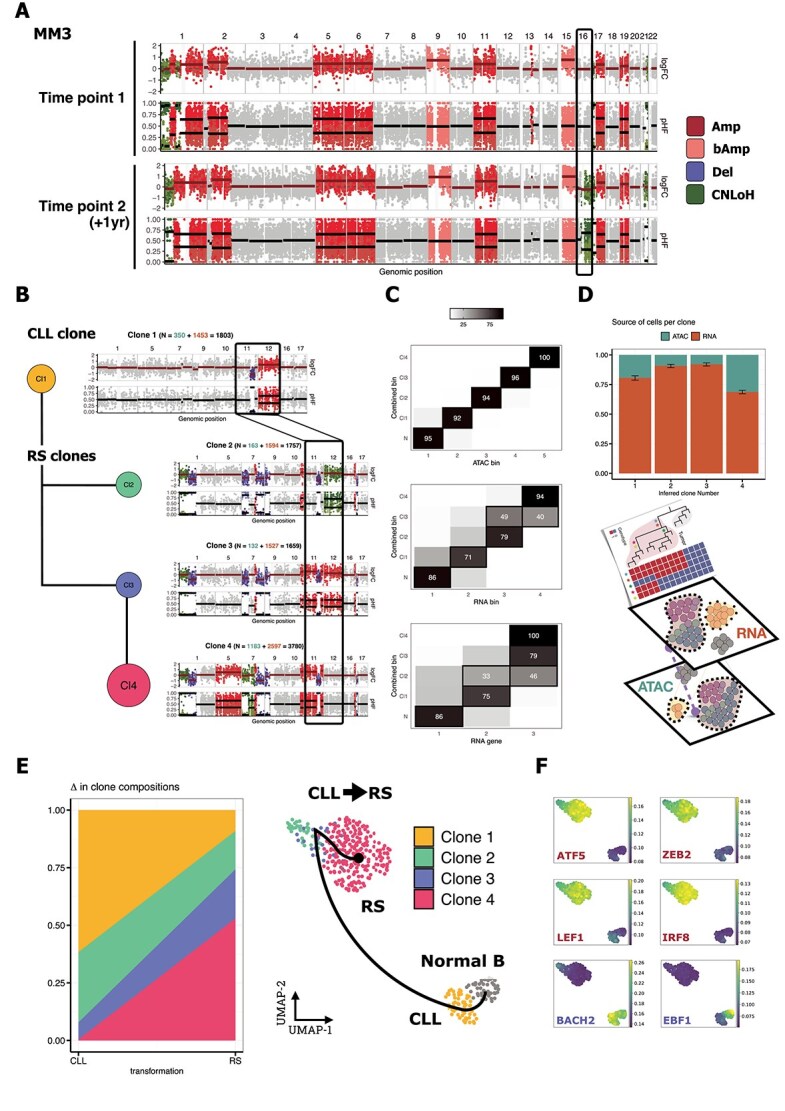
Numbat-multiome enabled accurate inference of complex clonal evolution from serial patient samples. (A) At the clone pseudobulk level, Numbat-multiome revealed an additional CNV in MM3_T2 (black rectangle) by summarizing logFC (top) and pHF (bottom) across two timepoints in the Combined bin mode Supplementary materials. (B) Clone profiles from four serial samples inferred with the Combined bin mode show clone sizes by cell counts, with modality-specific counts. (C) Heatmaps demonstrate clone assignment consistency across three modes compared with Combined bin, normalized by row. (D) RNA and ATAC composition per clone, with 95% confidence intervals for RNA cell proportions estimated by binomial tests. (E) Clone-constrained integration using SCENIC+ jointly embeds cells from both modalities. (F) Inferred gene regulatory networks revealed differential TF activity pre/post CLL-to-RS transformation (red = upregulated TF activities; blue = downregulated TF activities).

We assessed the ability of Numbat-multiome to detect new CNVs in serial samples from the same individuals using two approaches. First, we intentionally performed separate CNV inference for the two samples collected a year apart from the same MM patient (time point 1 in 2020 [MM3_T1] and time point 2 in 2021 [MM3_T2]). MM is a good model for studying cancer evolution due to its clinically well-defined precursor stage, SMM. MM and its precursor condition are often hyperdiploid, which is commonly diagnosed using Fluorescence In Situ Hybridization (FISH) data [27]. Hyperdiploidy is characterized by amplifications of multiple whole chromosomes, which could make CNV inference more challenging, as the average copy number is significantly higher than the usual two copies in normal cells. The higher ploidy can mislead methods to treat the diploid regions as deletions after normalizing by total coverage. Accurate CNV inference from nongenetic profiles (ATAC-seq and RNA-seq) of SMM can thus provide insights into the functional impact of CNV events and their candidate gene targets during MM development. We used Numbat-multiome to infer CNV profiles from the two MM samples (Fig. 3A) and found that they are overall consistent with each other. We were able to detect an additional CNV event in MM3_T2, a CNLoH of chr16q, using all four modes, confirming the robustness of our approach when applied to serially collected samples. As the clinical variables for this patient that are usually assessed during MM progression, including M-spike, free light chain ratio, and bone marrow plasma cell percentage, did not notably change (Supplementary methods) between the two time points, we cannot conclude whether CNLoH of chr16q was a driver event that increased the fitness of the cells, or was a passenger event carried by genetic drift. Nevertheless, the additional CNV event found in the second time point helped the reconstruction of the phylogenic tree for this patient.

Next, we tested whether aggregating several samples from the same patient within one Numbat-multiome run would reveal the existence of minor subclones prior to their detection by WGS. We used the CLL-to-RS paired samples as a test case for this approach. The genetic or epigenetic changes defining RS remain elusive; therefore, being able to isolate rare subclones and understand their clonal dynamics with a higher resolution has long been an unmet need of researchers in the field [28–30]. While WGS remains generally sensitive to CNV detection with improved algorithms, correctly inferring the co-occurrence and order of subclonal events in WGS remains challenging, and current methods cannot always confidently determine whether CNV events occurred in the same subclone. Using input data from cells obtained from four serial samples, Numbat-multiome identified rare subclones (clones 2 and 3) and inferred a phylogenetic tree with multiple CNV events occurring in the same genomic regions (chr11q and chr12) (Fig. 3B).

Running Numbat-multiome across our benchmark cohorts, the four different modes (RNA gene, RNA bin, ATAC bin, or Combined bin) generally yielded similar CNV estimates (Fig. 2A). In the case of more complicated clone structure with minor subclones (< 20% of cancer cells), the addition of ATAC-derived signals improved the accuracy of detecting subclonal CNV events and the assignment of cells to clones. The inference results of the ATAC bin were highly consistent with those from the Combined bin, while the two modes with only RNA-derived signals merged some of the subclones (Fig. 3C). Notably, when running Numbat-multiome in the Combined bin mode, each of four clones was supported both by scRNA- and scATAC-seq data, suggesting the signals were consistent across both data types, and that combining them enhanced the detection of subclones (Fig. 3D). We hypothesized that more abundant heterozygous SNP coverage from scATAC-seq data helped the inference of CNLoH events, which otherwise are challenging to infer using signals based on total molecule abundance. Therefore, adding scATAC-seq data to the inference process particularly improved the detection of the chr12 CNLoH event, resulting in the identification of the minor subclone, clone 2 (Supplementary Fig. S5).

Consistent CNV clone assignment and clustering of epigenomic profiles were generally observed across cells. This enabled the integration of gene expression and chromatin accessibility data in a clone-constrained manner [17]. The joint embedding of both profiles and our inferred step-wise clonal evolution allowed us to map an epigenetic trajectory for CLL-to-RS transformation (Fig. 3E).

An additional benefit of integrating RNA-seq with ATAC-seq data, and of using Numbat-multiome to identify clones, is the ability to study gene regulation and TF activities during clonal evolution. Indeed, our analysis revealed differential TF activities during the dynamic change from CLL to RS. In particular, we identified up-regulated TFs such as *ATF5*, *ZEB2*, *LEF1*, and *IRF8*, with previously unrecognized roles in RS pathogenesis, making them worthy of further investigation (Fig. 3F).

A key challenge in understanding RS biology is the incomplete elucidation of TFs driving transformation from CLL, largely due to difficulties isolating pure tumor populations and limited longitudinal patient specimens [28]. Recent studies have begun defining core transcriptional and epigenomic programs in RS transformation [12, 30], but our single-cell multi-omic approach is able to uniquely map dynamic TF activity within evolving clones.

In particularly, LEF1 (*LEF1*) functions as a prosurvival factor in CLL, and its knockdown leads to decreased leukemic cell viability [31–34]. Our analysis showed sustained *LEF1* activity within transformed RS clones. Although *LEF1*’s transcription was not reported higher at the time of transformation from previous gene expression studies [28, 30], recent immunohistochemical evidence indicates persistent LEF1 protein expression in RS, suggesting potential post-transcriptional regulation [35]. Our findings reinforce that sustained *LEF1* activity is likely important during transformation.

IRF8 plays a multifaceted role in CLL, RS, and DLBCL (the most common histology of RS). In CLL, elevated *IRF8* expression in hematopoietic stem cells suggests early epigenetic priming for leukemogenesis [36], and its cooperation with *PU.1* and *IRF4* is critical for suppressing malignant transformation in early B cells [37]. Additionally, IRF8 acts as a transcriptional activator of *CD37*, a therapeutic target in DLBCL, with its loss linked to poor prognosis [38]. Our analysis further highlights increased *IRF8* activity specifically in RS clones, providing direct single-cell evidence for its critical role as a transformation driver, consistent with recent genomic analyses identifying recurrent *IRF8* mutations and CNVs as key events in aggressive RS evolution [28, 30].

We also detected down-regulated TFs, including *BACH2* and *EBF1*, which have both been previously implicated as functionally important in CLL and RS (Fig. 3F). Low expression of the TF *BACH2* has been associated with adverse outcomes in CLL [39]. Our data confirm reduced *BACH2* activity within transformed RS clones, consistent with previous bulk transcriptomic analyses [28]. This supports the hypothesis that *BACH2* downregulation may contribute to RS transformation. This association was particularly notable in patients with mutated immunoglobulin heavy chain variable region and those harboring 11q or 13q Dels. These results collectively support the possible role of *BACH2* loss as a prognostic biomarker in CLL, potentially aiding in risk stratification and therapeutic decision-making. Reduced expression of *EBF1* in the DLBCL cell line KIS-1 disrupts B-cell-specific gene expression, while exogenous *EBF1* expression restores key B-cell programs such as *CD19* and *CD79b*, highlighting its potential as a therapeutic target to re-establish B-cell identity in RS [40].

Taken together, these findings demonstrate the capability of our single-cell multi-omic approach to dissect complex regulatory mechanisms in RS. By capturing TF dynamics at single-cell resolution, we provide valuable insights into transformation pathways, facilitating the development of precise mechanistic models and targeted therapeutic strategies for this aggressive malignancy.

## Conclusion

Our extension of Numbat to accommodate scATAC-seq and sample-paired multiome data broadens the scope and accuracy of haplotype-aware CNV inference in single-cell datasets. By leveraging complementary signals from RNA and accessible chromatin profiles, Numbat-multiome robustly and accurately infers CNVs and CNV-based phylogeny across both modalities, as well as in cases with several longitudinal samples per tumor. This enables the integration of scRNA- and scATAC-seq data in a clone-constrained manner and further investigations of the dynamic changes in transcription, usage of regulatory elements, and TF activities. These findings underscore the value of integrated single-cell analyses for studying tumor evolution and highlight the potential of Numbat-multiome as a general tool for high-resolution clonal characterization in future cancer research.

**Key Points**

- **Innovative Methodology**: Introduction of a unified multiomic binning strategy and integration of RNA-seq and ATAC-seq data for haplotype-aware copy number variation inference.
- **Enhanced Clonal Resolution**: Accurate detection and tracking of subclonal events and tumor evolution over time, even in complex multi-sample studies.
- **Biological Insights from Integrative Analysis**: Ability to integrate transcriptional and epigenetic data at the single-cell level, providing novel insights into regulatory landscapes and evolutionary trajectories underlying cancer progression.

## Supplementary Material

suppFig1_bbaf516

suppFig2_bbaf516

suppFig3_bbaf516

suppFig4_bbaf516

suppFig5_bbaf516

suppFig6_bbaf516

Numbat_multiome_25_09_09-SuppTable_bbaf516

Numbat_multiome_25_09_09-SuppMethods_bbaf516

## Data Availability

A step-by-step walkthrough of preparing inputs and running Numbat-multiome is provided as a vignette in the original Numbat github repo at https://kharchenkolab.github.io/numbat/articles/numbat-multiome.html. The analysis scripts used to generate the results in this paper are available at https://github.com/getzlab/Numbat-multiome_Analysis. A stand-alone docker image containing a snakemake workflow as well as the required dependencies for running Numbat-multiome with raw inputs (count matrix, fragments table, and aligned BAM files) are available at: gcr.io/broad-getzlab-workflows/numbat-multiome:latest
