# Supplementary material for: Numbat-multiome: inferring copy number variations by combining RNA and chromatin accessibility information from single-cell data
Source: Brief Bioinform. 2025 Oct 17;26(5):bbaf516. doi: 10.1093/bib/bbaf516 (PMC12531993; doi:10.1093/bib/bbaf516)
Supplement: Numbat_multiome_25_09_09-SuppTable_bbaf516 [file numbat_multiome_25_09_09-supptable_bbaf516.pdf]

mode	precision			
	mean	median	q25	q75
RNA gene	0.976	0.975	0.970	0.982
RNA bin	0.970	0.975	0.970	0.985
ATAC bin	0.974	0.985	0.955	0.990
Combined bin	0.980	0.985	0.978	0.990

(a) Summary statistics for precision

mode	recall			
	mean	median	q25	q75
RNA gene	0.973	0.990	0.980	0.990
RNA bin	0.971	0.980	0.960	0.990
ATAC bin	0.973	0.985	0.978	0.990
Combined bin	0.974	0.985	0.980	0.990

(b) Summary statistics for recall

mode	F1			
	mean	median	q25	q75
RNA gene	0.974	0.980	0.970	0.990
RNA bin	0.970	0.980	0.970	0.982
ATAC bin	0.973	0.980	0.970	0.990
Combined bin	0.978	0.990	0.980	0.990

(c) Summary statistics for F1

Table 1: Supplementary Table S1: Performance metrics for all models.
