# Supplementary material for: Numbat-multiome: inferring copy number variations by combining RNA and chromatin accessibility information from single-cell data
Source: Brief Bioinform. 2025 Oct 17;26(5):bbaf516. doi: 10.1093/bib/bbaf516 (PMC12531993; doi:10.1093/bib/bbaf516)
Supplement: Numbat_multiome_25_09_09-SuppMethods_bbaf516 [file numbat_multiome_25_09_09-suppmethods_bbaf516.pdf]

Supplementary Methods

scRNA-seq and scATAC-seq data processing

scRNA-seq filtering for CLL-to-RS case: We filtered the feature-barcode gene expression matrix to keep only high quality cells (>1000 total counts, >300 detected genes and <10% of mitochondrial reads) and expressed genes (detected in $\geq$ 3 cells). Downstream analyses were performed using Scanpy [33]. Doublet removal was performed and predicted doublets with Scrublet[41] by comparing to simulated distribution was removed (N = 503). We applied total normalization followed by log1p transformation to cell-by-gene matrix. The top 6000 most variable genes (with hemoglobulin, mitochondrial, ribosomal and immunoglobulin genes, N=253) was used to perform principal component analysis. The top 50 PCs were further used for Louvain graph-based clustering and UMAP embedding visualization. Sample IDs were used as the batch key for harmony correction on the top 50 PCs. The harmony-corrected PCs were further used for downstream analysis.

scATAC-seq analysis for CLL-to-RS case: scATAC-seq data were processed using the pycisTopic framework [42]. Fragment files were grouped into pseudo-bulk profiles by sample ID, and consensus peaks were called using MACS2 [43] with BEDPE input format, –shift 73, –extsize 146, and –qvalue 0.05. Peaks overlapping the hg38 ENCODE blacklist were excluded. Quality control metrics were computed per barcode, including TSS enrichment, unique fragment count, and FRiP. Barcodes were retained if they passed automatically inferred thresholds (TSS enrichment >13.3; unique fragments >1,300). Doublets were identified and filtered using Scrublet [41] with an expected doublet rate of 10% and a decision threshold of 0.3.

LDA topic modeling was performed via Mallet across 11 topic numbers (2–50), selecting the final model using coherence and log-likelihood. Cell clustering was performed using the Leiden algorithm (k=10, resolutions=[0.6, 1.2, 3.0]) with batch correction via Harmony on sample pools. Topic distributions were binarized using Otsu or Li thresholding, and differential accessible regions (DARs) were identified using log2FC > log2(1.5) and FDR < 0.05. Gene activity was inferred using weighted region aggregation across gene bodies and flanks (upstream 1–100 kb, downstream 1–100 kb) with distance-decay weighting and optional Gini scaling.

Identification of immune cells: The three main classes of non-B immune cells present in this cohort are: T cells; monocytes and erythrocytes. Expression of markers (erythrocyte: CRHBP, CA1, GATA1; monocyte: FCER2, S100A9, MS4A7, S100A12; T cell: IL7R, PRF1 and NKG7) per RNA-cluster across patient samples were examined and further separated from B cells. Similarly, the same marker were used to identify immune clusters in scATAC-seq data based on marker's promoter chromatin accessibility or derived gene activity calculated based on TSS-extended regions.

Additional WGS Threshold Sensitivity Analysis

To validate our default thresholds for amplified ($\geq$2.5 copies) and deleted ($\leq$1.3 copies) genomic regions, we evaluated multiple cutoffs (2.3, 2.4, 2.6, and 2.7 for amplifications;

1.4, 1.5 for deletions). Precision, recall, and F1 varied moderately, but the overall trends remained consistent.

Clinically used biochemical variables for myeloma diagnosis

The 20/2/20 criteria[44] is a risk stratification model used to assess the risk of progression in SMM to overt MM. It's based on three factors: bone marrow plasma cells (BMPC) >20%, serum M-protein > 2 g/dL, and the involved/uninvolved free light chain (FLC) ratio > 20. Patients are categorized into low, intermediate, and high-risk groups based on the number of these risk factors present.

Furthermore, expanded beyond the standard CRAB (Calcium elevation, Renal failure, Anemia, and Bone lesions) features of myeloma, the International Myeloma Working (IMWG) updated the diagnostic criteria for overt MM to include specific biomarkers indicative of disease progression[45]. These criteria encompass: (i) clonal BMPC $\geq$ 60% (ii) FLC ratio ≥ 100; and (iii) presence of > 1 focal lesion on magnetic resonance imaging in patients having bone marrow with at least 10% of clonal BMPC infiltration.

For two serial samples from the same patient MM3, based on the available clinic data: BMPCs remains 70%; serum M-protein increased from 2.3 to 3g/dL,; and FLC increased from 73 to 76. According to IMWG updated criteria, both samples should be classified as overt MM (clonal BMPC $\geq$ 60%). The other two biochemical variables, serum M-proteins and FLC ratio, collected from those two serial samples are also unambiguously higher than the risk stratification criteria (15% and 50% higher for M-protein; 265% and 280% higher for FLC ratio).
